# Enhancing Cerebral Blood Flow Quantification: A Comprehensive Review of Denoising, Artifact Correction, and Simulation in Arterial Spin Labeling MRI

**DOI:** 10.3390/s26165202

**Published:** 2026-08-17

**Authors:** Shyna A, Jini Raju, Ansamma John, Chandrasekharan Kesavadas, Aditya Ajith, Manu J. Pillai, Shameem Ansar, Ginu Rajan

**Affiliations:** 1 Department of Computer Science and Engineering, Thangal Kunju Musaliar College of Engineering, APJ Abdul Kalam Technological University, Kollam 691005, India; s4shyna@gmail.com (S.A.);; 2 Department School of Computer and Information Science, Vidya Academy of Science and Technology Technical Campus, APJ Abdul Kalam Technological University, Trivandrum 695602, India; 3 Department of Imaging Sciences and Interventional Radiology, Sree Chitra Tirunal Institute of Medical Sciences and Technology, Trivandrum 695011, India; 4 Centre for Engineering Research in Intelligent Sensors and Systems, Cardiff School of Technologies, Cardiff Metropolitan University, Cardiff CF5 2YB, UK

**Keywords:** arterial spin labeling MRI, cerebral blood flow, machine learning, deep learning, artifacts, dataset simulation

## Abstract

Arterial Spin Labeling (ASL) Magnetic Resonance Imaging (MRI) is a noninvasive imaging technique used to quantify cerebral blood flow (CBF) by using magnetically labeled arterial blood water as an endogenous tracer. Although ASL eliminates the need for exogenous contrast agents, its widespread clinical use is limited by several challenges, including low Signal-to-Noise Ratio (SNR), susceptibility to motion, and various imaging artifacts. To address these limitations, both traditional denoising techniques and Machine Learning (ML)/Deep Learning (DL)-based approaches have been developed to improve the reliability of ASL by reducing noise, correcting artifacts, and enhancing image quality. In addition, the generation of simulated ASL datasets has become an important strategy for training and validating novel methods when sufficient clinical data are unavailable. This review examines conventional image-processing techniques together with modern machine learning and deep learning approaches developed to improve ASL image quality through denoising and enhancement. It also discusses the major artifacts that affect ASL acquisition and summarizes the simulation methodologies used for the development and evaluation of new algorithms.

## 1. Introduction

Arterial Spin Labeling (ASL) Magnetic Resonance Imaging (MRI) [1,2] is a quantitative perfusion imaging modality widely used to measure cerebral blood flow (CBF). Unlike contrast-enhanced perfusion methods [3,4,5], ASL relies on inverting the magnetization of inflowing blood at a labeling plane using an appropriate labeling scheme such as Continuous ASL (CASL), Pulsed ASL (PASL) and pseudo-continuous ASL (PCASL) schemes [6,7,8,9,10]. During this process, Radio Frequency (RF) pulses are applied for a specified labeling duration (τ), after which the labeled blood requires a finite travel time, referred to as Arterial Transit Time (ATT) [11], to reach the tissue. To account for this, a Post-Labeling Delay (PLD) is introduced before image acquisition, and in some cases, multi-PLD acquisitions are employed to capture regional variations in ATT and improve quantification accuracy. In practice, ASL involves acquiring both label images, in which blood is inverted, and control images, acquired without labeling. Subtracting the label from the control produces the Perfusion-Weighted Image (PWI), which reflects tissue perfusion but suffers from an intrinsically low Signal-to-Noise Ratio (SNR). To mitigate this, ASL experiments are typically repeated 20–40 times [6], generating multiple Label–Control (L–C) pairs at relatively low spatial resolution, followed by averaging of the subtracted images to create a stable PWI. This image can then be converted into a CBF map using the Buxton kinetic model [12].

Despite its clinical potential, the reliability of ASL remains constrained by various acquisition-related artifacts, specifically Partial Volume Effects (PVE) [13]. PVEs occur when a voxel contains mixed gray matter (GM), white matter (WM), and cerebrospinal fluid (CSF), leading to biased CBF estimation. While conventional denoising, PVE correction, and artifact reduction strategies have been proposed, recent advances in Artificial Intelligence (AI) offer new opportunities to enhance ASL data quality and quantification accuracy.

Over the past two decades, several review articles have been published in the field of ASL MRI, discussing acquisition principles, CBF quantification models [14,15], partial volume correction [16], and clinical applications [17,18,19,20,21,22,23,24,25]. A comparative summary of these review articles is presented in Table 1. It is evident that most existing reviews primarily focus on the fundamentals of ASL and its clinical applications. In recent years, machine learning (ML) and deep learning (DL) have emerged as promising approaches for improving ASL image quality. However, to the best of our knowledge, no previous review has comprehensively examined AI-based denoising, artifact and outlier correction, simulation frameworks, and their combined role in enhancing CBF quantification and facilitating the clinical translation of ASL MRI.

While artifact correction is a recurring topic in MRI literature, it is often addressed only briefly in ASL reviews [26,27]. Furthermore, simulation-based ASL datasets constitute an essential resource for generating training material and benchmarking algorithms in the absence of large, high-quality clinical datasets. A detailed exposition of the procedures underlying their generation is therefore crucial to guide researchers newly entering the ASL domain.

These emphases raise several research questions for this review:

RQ1. Denoising methods—How have traditional and AI-driven denoising strategies improved the robustness of ASL data, and what challenges remain?

RQ2. Artifact impact—What are the most common acquisition-related artifacts in ASL, and how do they directly affect the accuracy and reliability of CBF quantification?

RQ3. Simulation role—In what ways can simulation-based ASL datasets not only complement or substitute clinical data but also serve as practical guidance for beginners in generating training and benchmarking material for algorithm development?

RQ4. Clinical translation: What are the existing challenges and future opportunities for ASL MRI in clinical practice, particularly concerning standardization, reproducibility, and integration with advanced computational and imaging techniques?

By addressing these questions, this review aims to clarify the current state of AI-enhanced ASL post-processing, highlight the challenges posed by acquisition-related artifacts, examine the role of simulation datasets in method development, and provide a roadmap for future research in improving CBF quantification and moving ASL MRI closer to clinical translation.

To ensure a comprehensive overview of the field, the literature included in this review was identified through searches of major scientific databases, including PubMed, Scopus, IEEE Xplore, Web of Science, and Google Scholar. A combination of keywords related to ASL, CBF), denoising, artifact correction, simulation, machine learning, deep learning, and super-resolution was used to identify relevant publications. While greater emphasis was placed on recent studies, landmark contributions were also included to provide the necessary background and context. The selected articles were chosen based on their relevance to the objectives of this review and their contribution to advances in ASL image enhancement and CBF quantification.

The rest of the paper is organized as follows: Section 2 presents an overview of different methods developed to improve CBF quantification with minimal L-C pairs, focusing on RQ1. Section 3 focuses on RQ2, examining the different types of artifacts encountered during ASL image acquisition and their effects on the reliability of CBF quantification. Section 4 investigates RQ3, discussing the role of dataset simulation in overcoming the absence of clinical data and ensuring robust ASL MRI model development. Section 5 addresses RQ4 by reviewing the primary challenges and future prospects in ASL MRI. Finally, Section 6 outlines the conclusions drawn from this review.

## 2. ASL Image Enhancement

**Figure 1:**
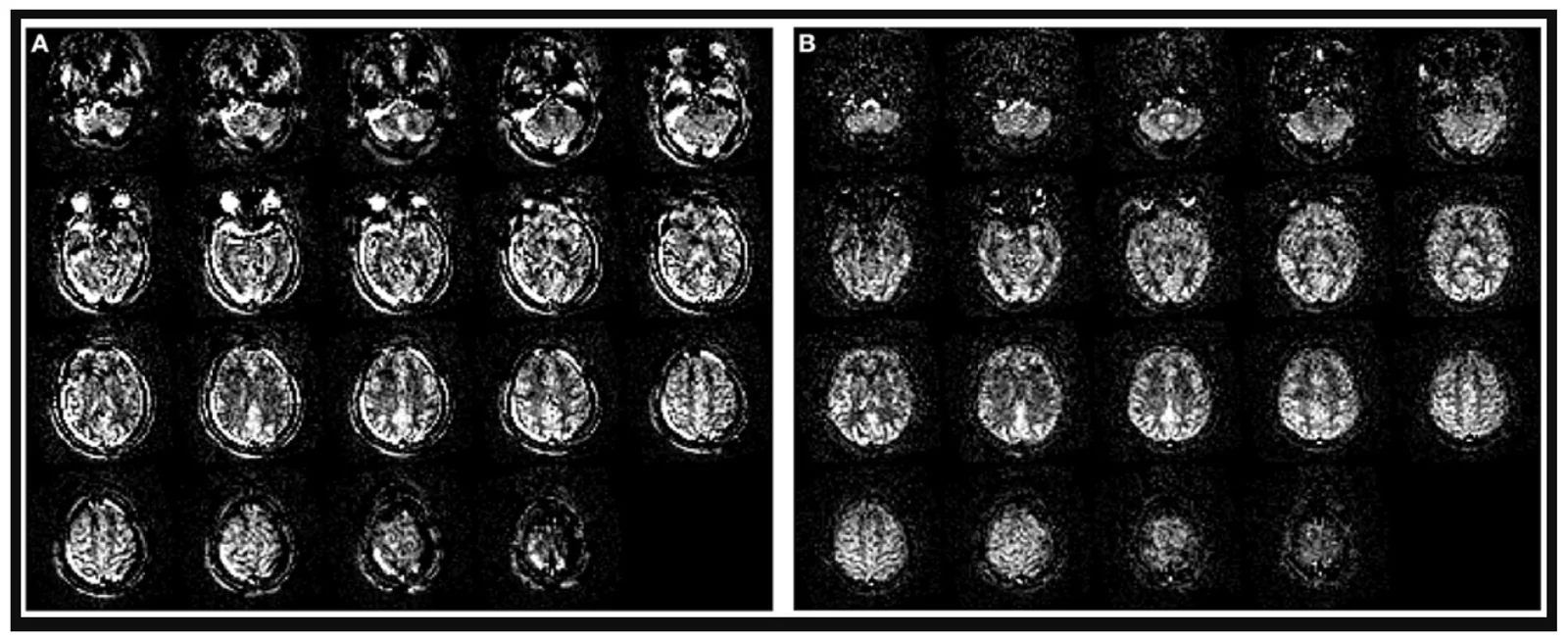
The effect of motion correction. (**A**) Perfusion image without motion correction; (**B**) The same perfusion image after motion correction. Motion artifacts can be easily recognized by prominent patterns of very high and very low (i.e., negative) signals next to each other in areas where the static tissue has high contrast differences—visible as rims around the brain edge and skull [17]. [Reprinted from Frontiers in Radiology, Clement P et al., “A Beginner’s Guide to Arterial Spin Labeling (ASL) Image Processing,”, 2, 929533 (2022), doi: 10.3389/fradi.2022.929533, under the terms of the Creative Commons Attribution 4.0 International License (CC BY 4.0)].

The challenges associated with low SNR in ASL MRI have encouraged the research community to improve the accuracy of CBF quantification. The typical ASL perfusion signal is merely 1% of the total MRI signal [9,25], necessitating the acquisition of multiple L-C pairs and subsequent averaging to create PWI. While this averaging mitigates the impact of noise, it leads to extended scan times, patient discomfort, and motion artifacts, presenting a significant impediment to the widespread clinical adoption of ASL imaging. Motion correction is an essential preprocessing step in ASL image analysis and is routinely performed before denoising and CBF quantification. By correcting patient motion and improving the spatial alignment of successive L–C pairs, it minimizes motion-induced artifacts and provides more reliable inputs for subsequent machine learning- and deep learning-based denoising methods. In practice, this preprocessing step is commonly implemented using established software packages such as Statistical Parametric Mapping (SPM), Functional Magnetic Resonance Imaging of the Brain Software Library (FSL) and Advanced Normalization Tools (ANTs). Figure 1 depicts the effect of motion correction on image quality.

The ASL research community seeks improvements in the SNR of PWI or CBF maps to enable accurate CBF estimation without the need for acquiring numerous L-C pairings, whether through traditional approaches or AI-based methods.

**Figure 2:**
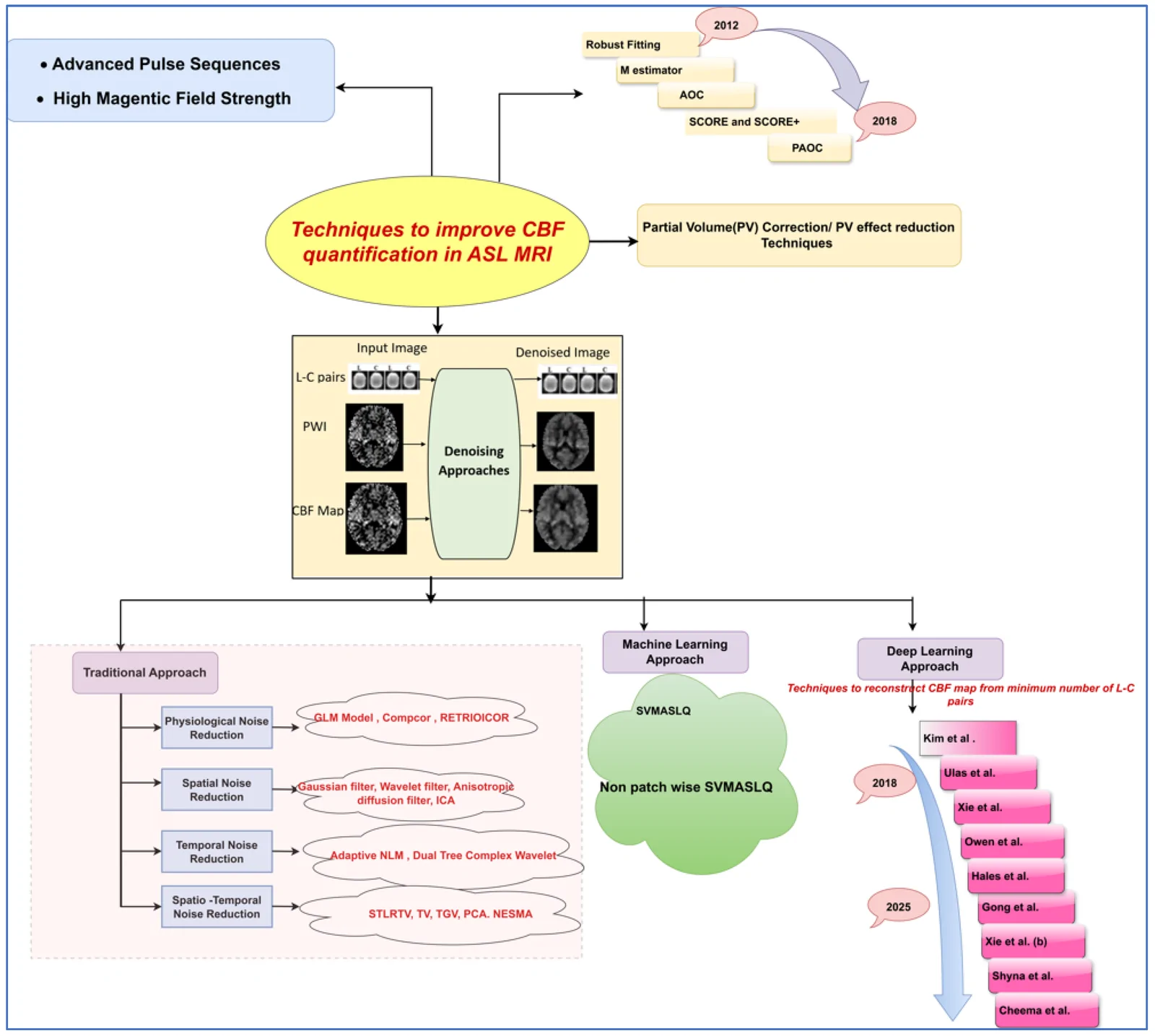
Illustration of existing techniques to improve CBF quantification in ASL MRI. The figure provides an overview of the major approaches reported in the literature to enhance the accuracy and quality of CBF estimation. These include advanced pulse sequence strategies [28,29,30,31,32], artifact and outlier removal methods, illustrating the chronological development from robust fitting [33] and M-estimator methods [34] to AOC [35], SCORE/SCORE+ [36], and PAOC [37] techniques; partial volume (PV) correction methods [16] for reducing tissue-mixing effects; and denoising approaches [38,39,40,41,42,43,44,45,46,47,48,49,50,51,52,53,54,55,56,57,58,59,60]. The denoising methods are further categorized into traditional approaches [38,39,40,41,42,43,44,45,46,47,48,49], machine learning-based approaches [50,51] and deep learning-based approaches [52,53,54,55,56,57,58,59,60], highlighting the evolution of neural network models developed for reconstructing high-quality CBF maps from a limited number of L–C pairs.

Figure 2 represents the overview of the techniques used to improve the CBF quantification in ASL MRI.

### 2.1. Traditional Methods for Improving the Quality of ASL Images

**Figure 3:**
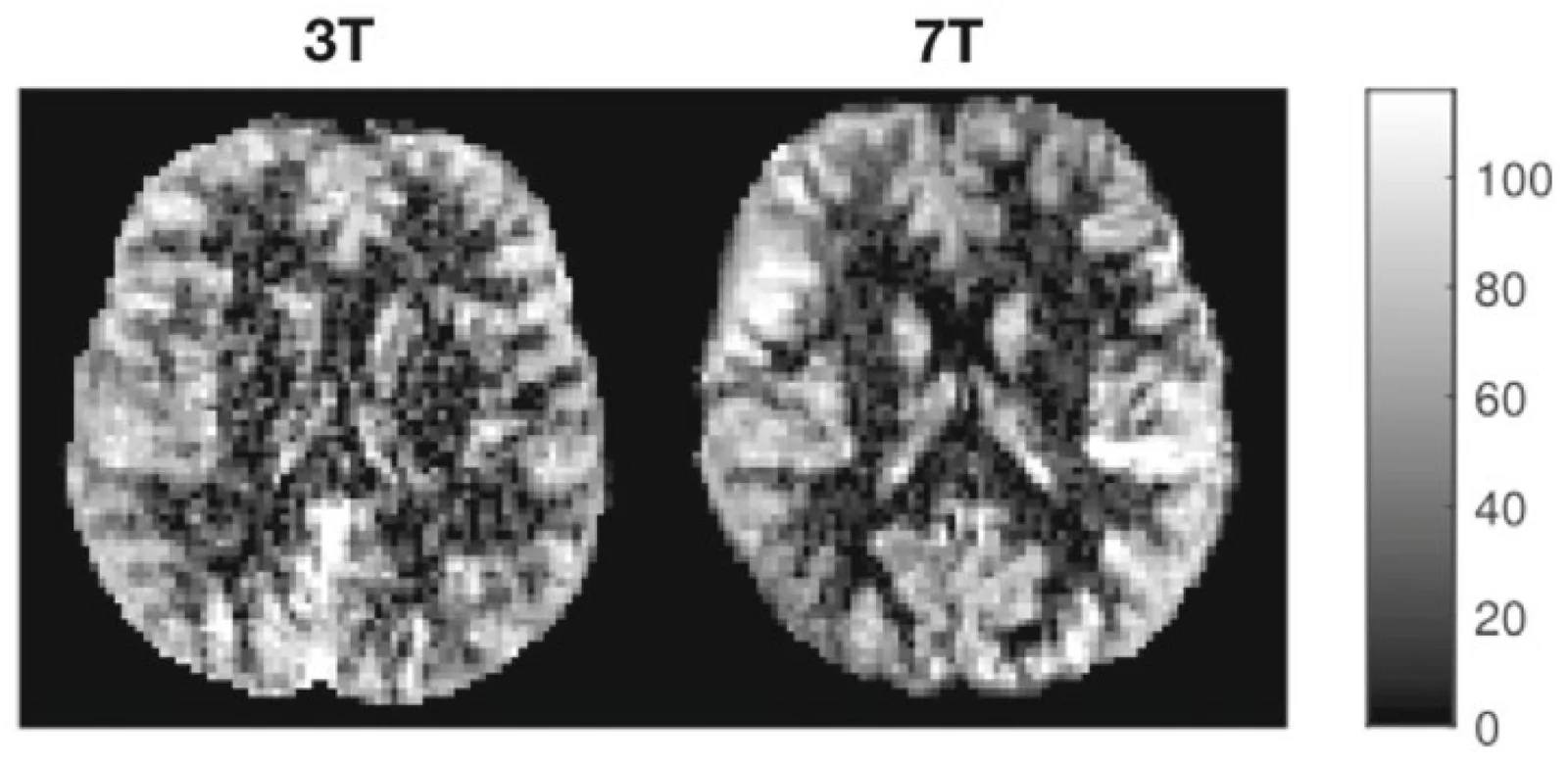
Effect of magnetic field strengths on CBF map [32]. [Reprinted from Magnetic Resonance in Medicine, Hernandez-Garcia L et al., “Recent technical developments in ASL: a review of the state of the art,” 88, 2021–2042, (2022), doi: 10.1002/mrm.29381, under the terms of the Creative Commons Attribution 4.0 International License (CC BY 4.0)].

Researchers have addressed the challenges in CBF quantification through a variety of strategies. One approach focuses on developing advanced techniques that employ different pulse sequences and higher magnetic field strengths [28,29,30,31,32]. Figure 3 shows the ASL CBF map generated in the same subject using the same protocol at 3T and 7T.

It is clear from Figure 3 that the image generated using 7T is less noisy compared to 3T. However, the advantages of higher field strength are accompanied by several practical and technical challenges, including increased magnetic field inhomogeneity, greater susceptibility to artifacts, higher Specific Absorption Rate (SAR), and the need for specialized hardware and advanced pulse sequence design. In addition, the limited availability of ultra-high-field MRI systems restricts their routine use in many clinical settings. In an effort to improve the quality of ASL images, many researchers have proposed a variety of post-processing techniques to improve the accuracy of CBF quantification in ASL MRI. These approaches include noise reduction, compensation for tissue mixing caused by Partial Volume Effects (PVE) [16], and artifact and outlier correction. Among these, PVE is a major source of error arising from the relatively low spatial resolution of ASL, where individual voxels often contain a mixture of gray matter, white matter, and cerebrospinal fluid, resulting in biased perfusion estimates. Over the years, numerous PVE correction methods have been developed, including regression-based [61], Bayesian [13], and joint estimation approaches [62] that combine structural MRI information with ASL data to improve tissue-specific CBF estimation. Comprehensive reviews of these correction strategies are already available in the literature, particularly the review by Chappell et al. [16]. Therefore, rather than duplicating the existing literature, the remainder of this section focuses on complementary post-processing approaches, including noise reduction, artifact and outlier correction, and AI-based image enhancement techniques that further improve the quality and reliability of ASL-derived CBF measurements.

#### Noise Reduction—Traditional, Machine Learning and Deep Learning Techniques

The effectiveness of noise reduction strategies in ASL MRI largely depends on understanding the nature of noise present in MR images. Many of the traditional denoising methods used in ASL MRI originate from conventional MRI denoising; however, they have been adapted to address the unique characteristics of ASL imaging, including its inherently low SNR, physiological fluctuations, and the subtraction-based generation of perfusion-weighted images. Noise in MR data typically follows a Rician distribution [63,64], meaning that each label and control image can be modeled accordingly. Since ASL signals are obtained from the subtraction of L–C image pairs, the resulting PWI inherit noise characteristics that can be approximated by a Gaussian distribution [41]. This makes ASL data particularly well-suited for noise reduction methods that assume Gaussian or white noise models. However, in addition to Gaussian noise, physiological fluctuations caused by cardiac and respiratory cycles further degrade signal precision and complicate the quantification of CBF [40]. Table 2 summarizes different strategies proposed for noise reduction in ASL MRI, grouped into physiological noise correction [38,39,40], reduction in spatial noise [41,42], temporal noise [43,44] and spatio-temporal noise [45,46,47,48,49], ML techniques [50,51] and DL techniques [52,53,54,55,56,57,58,59,60]. Each method is outlined with its principle, advantages and limitations, providing a comparative perspective on how noise suppression has been approached from multiple angles.

From the comparison, it is clear that noise reduction in ASL MRI has progressed from simple model-based and filtering techniques toward more sophisticated spatio-temporal, ML and DL approaches. Early methods addressed either physiological noise or improved SNR through local filtering, but often compromised on edge sharpness or temporal fidelity. Spatio-temporal strategies provided a better balance between denoising and preservation of structural/physiological details, though with greater computational demands. Recent ML and DL methods achieve stronger denoising and edge preservation, but face challenges in terms of dataset availability and generalizability. Table 3 provides a detailed account of individual methods reported in the literature. This table emphasizes the diversity of methodological innovations and their relative strengths and limitations in improving CBF quantification. The reviewed studies demonstrate that acquisition parameters and labeling strategies critically influence the quality and reliability of CBF quantification in ASL MRI. Most physiological and spatial noise reduction studies employed single PLD or single Inversion Time (TI) acquisitions, while temporal and spatio-temporal methods often utilized multi-TI or multiple PLD schemes to capture dynamic perfusion information.

The number of L–C pairs varied widely across studies, from as few as 3–5 pairs in simulation-based evaluations to over 500 pairs in high-fidelity human experiments, reflecting the trade-off between scan time and SNR. Labeling techniques such as Proximal Inversion with a Control for Off-Resonance Effects (PICORE), CASL, PASL, and PCASL [10] were selected based on experimental goals, with PCASL being preferred in recent machine learning and deep learning applications due to its robustness and higher SNR [10]. Evaluation metrics consistently included Mean Squared Error (MSE), SNR, Contrast to Noise Ratio (CNR), Peak SNR (PSNR), Structural Similarity Index (SSIM), and statistical measures like t-tests or Bland–Altman analysis, emphasizing the importance of both quantitative fidelity and clinical interpretability.

Overall, the findings highlight that optimizing PLD/TI selection, L–C pair numbers, and labeling schemes, along with robust performance evaluation, is essential for accurate and reproducible CBF measurements, particularly in advanced ML/DL-based denoising frameworks.

**Figure 4:**
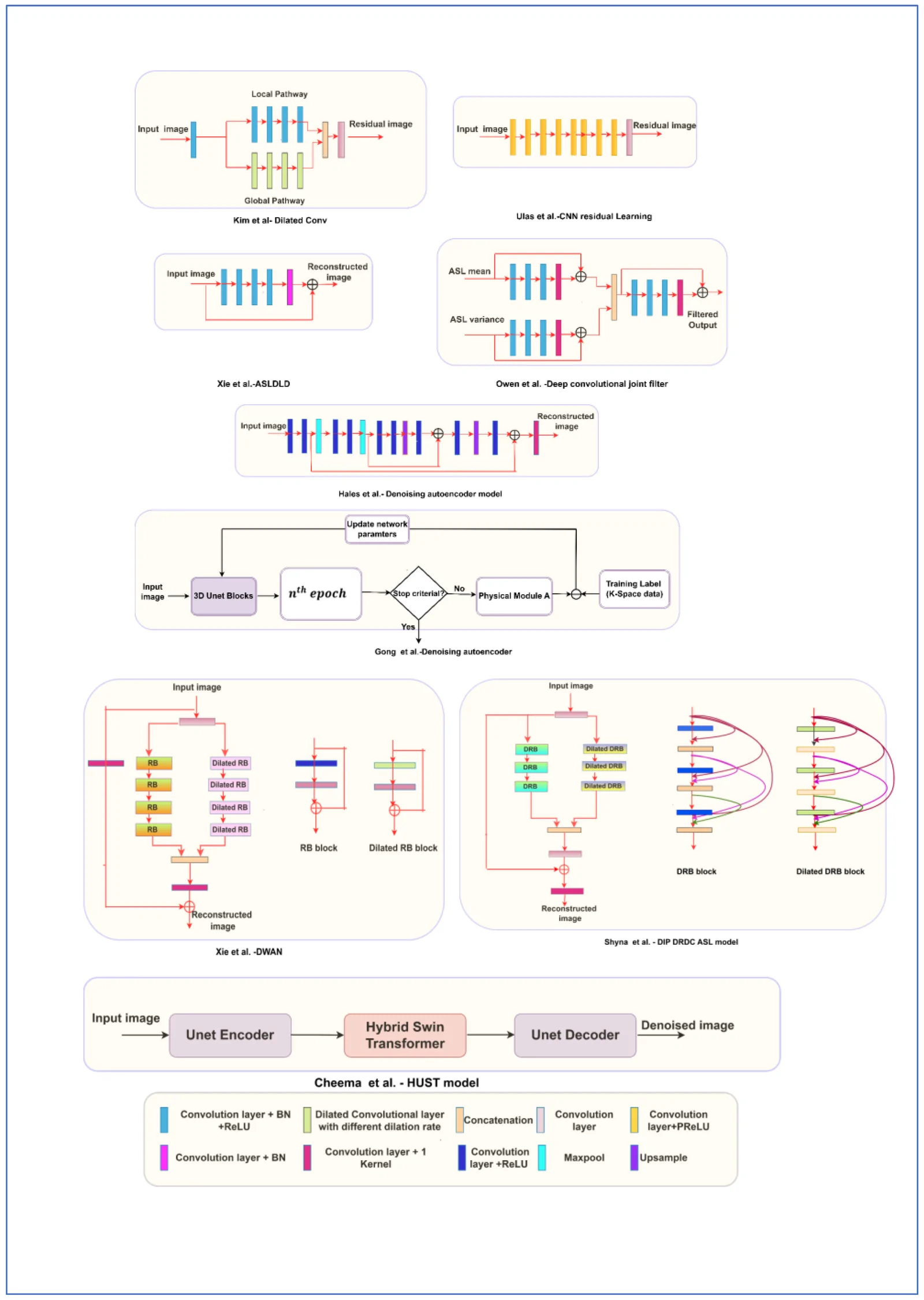
Overview of different deep learning models to improve CBF quantification in ASL MRI. The figure summarizes representative deep learning architectures proposed for ASL image denoising, reconstruction, and CBF map enhancement. It includes the dilated convolution network proposed by Kim et al. [52], the residual CNN by Ulas et al. [53], the ASLDLD model by Xie et al. [54], the deep convolutional joint filter by Owen et al. [55], the denoising autoencoder by Hales et al. [56], the physics-guided deep learning framework by Gong et al. [57], the Dilated Wide Activation Network (DWAN) by Xie et al. [58], the proposed Dual Independent Pathway–Densely Connected Residual Network with Dilated Convolution (DIP-DRDC) model by Shyna et al. [59], and the recent Hybrid Swin Transformer U-Net (HUST) model by Cheema et al. [60]. The figure also provides the legend of the convolutional, residual, dilated, pooling, upsampling, and concatenation blocks used in the network architectures.

Deep learning, particularly Convolutional Neural Network (CNNs) and their variants, has emerged as the dominant tool for ASL MRI denoising due to its ability to learn hierarchical features automatically, offering robustness against scale, orientation, and illumination variations. These models have been applied for denoising [65,66], reconstruction [59], and artifact removal, ultimately aiming to improve CBF quantification from fewer L–C pairs, thereby reducing acquisition time. A variety of network designs—ranging from residual learning, dual-pathway CNNs to wide-activation networks and Transformer-based models—have been explored with promising results across clinical and synthetic datasets as shown in Figure 4.

From the comparative analysis of deep learning approaches in ASL MRI denoising, it is evident that dual-pathway CNNs and wide-activation networks provide strong generalization capabilities, particularly when trained on clinical datasets, whereas residual learning strategies achieve high accuracy but remain dependent on the availability of clean reference images. Although simulated datasets are commonly used for training due to the scarcity of clinical data, validation on real patient cohorts is still essential to establish clinical utility. Most studies focus on PASL or PCASL perfusion images, occasionally supplemented with structural priors like T1-weighted Magnetization-Prepared Rapid Gradient Echo (MPRAGE), indicating the importance of multi-modal integration. Despite the consistent improvements over classical methods, several challenges persist, including the limited availability of large, diverse clinical datasets, the reliance on simulated data that may not fully capture clinical variability, and reduced generalizability when models trained on healthy volunteers are applied to pathological conditions. Furthermore, many supervised methods still require clean ground-truth CBF maps, which are difficult to obtain in practice. While spatio-temporal approaches show promise in tackling artifacts, robust solutions for motion- and acquisition-related noise remain underexplored. Overall, deep learning-based denoising demonstrates significant potential for improving cerebral blood flow quantification in ASL, but translation into routine clinical workflows demands broader datasets, improved robustness to pathology, and reduced dependence on reference data.

The methods discussed above primarily focus on reducing noise in ASL raw images, PWIs, or reconstructed CBF maps. ASL denoising can be performed either after CBF reconstruction or directly on the raw L–C pairs before CBF quantification. Most existing methods operate on reconstructed CBF maps, whereas denoising L–C pairs before CBF estimation has the potential to reduce noise propagation and improve quantification accuracy. However, this approach requires access to raw ASL data and has received relatively limited attention. Further studies are therefore needed to evaluate the effectiveness of these two strategies. Beyond noise, outliers and artifacts can impact the accuracy and reliability of perfusion data. Hence, the researchers focused on developing new methods and algorithms for reducing the artifacts and outliers in ASL.

## 3. Artifacts and Outlier Removal

**Figure 5:**
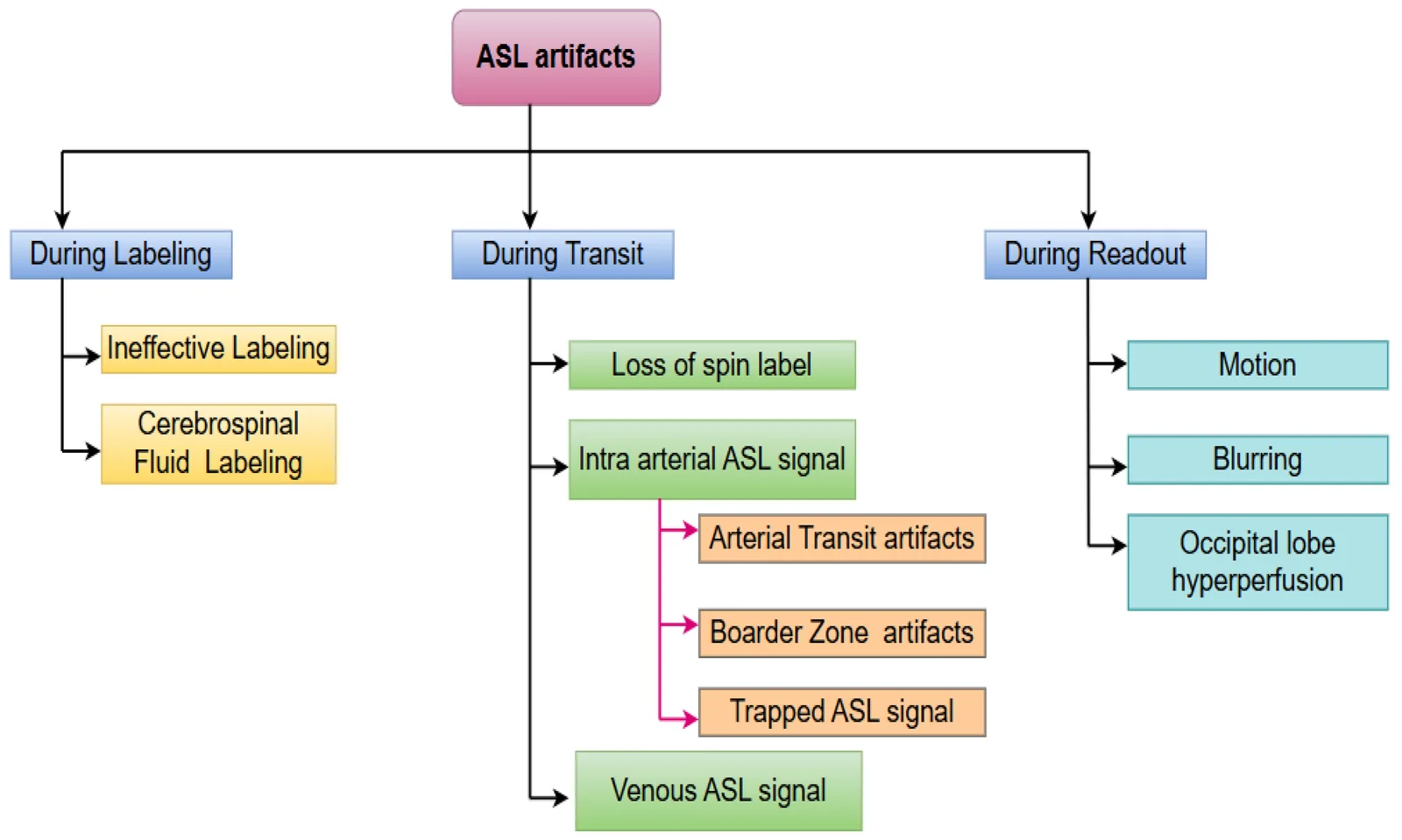
Overview of the different artifacts occurring during the ASL image acquisition process.

**Figure 6:**
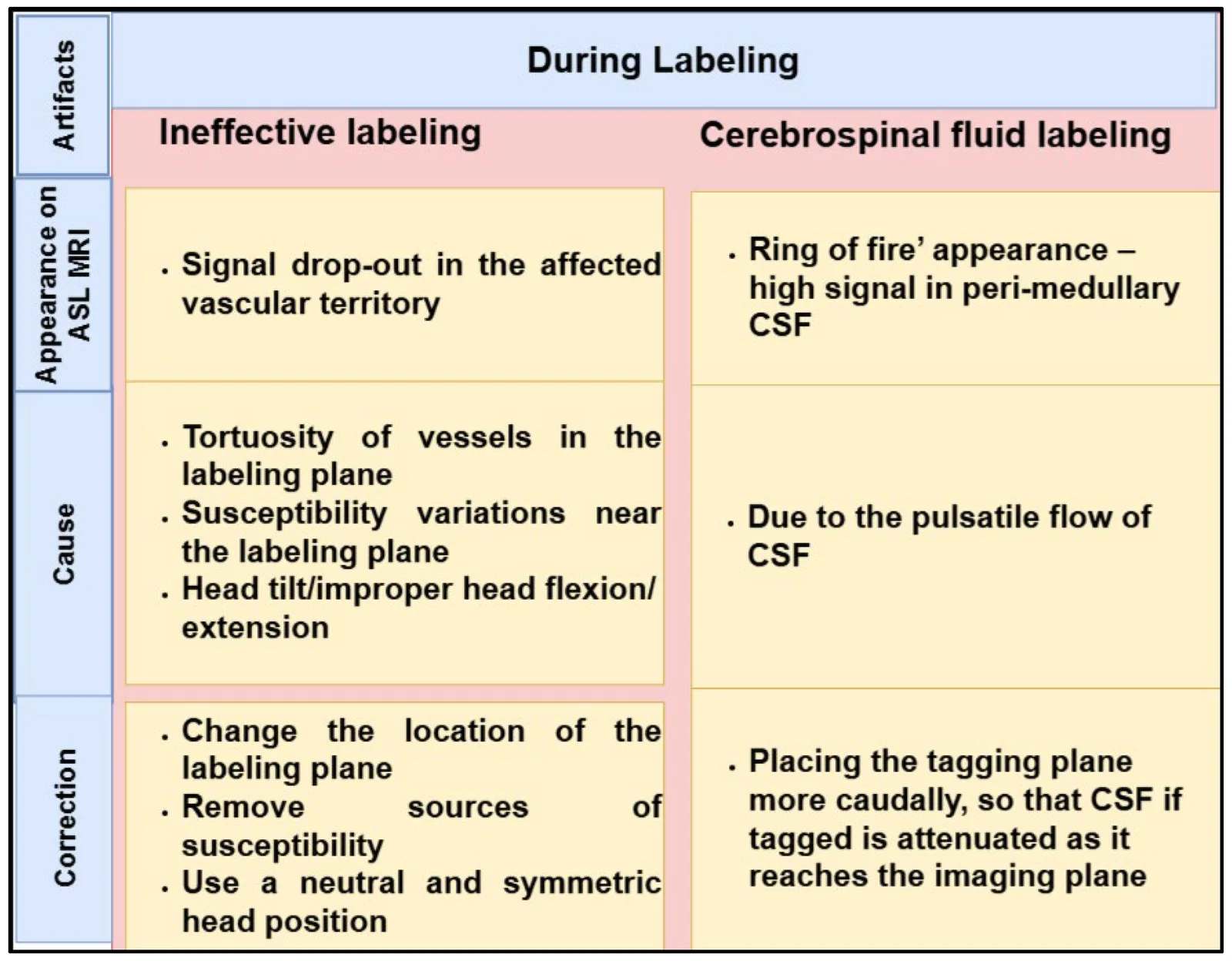
Artifacts occurring during blood labeling in ASL MRI.

**Figure 7:**
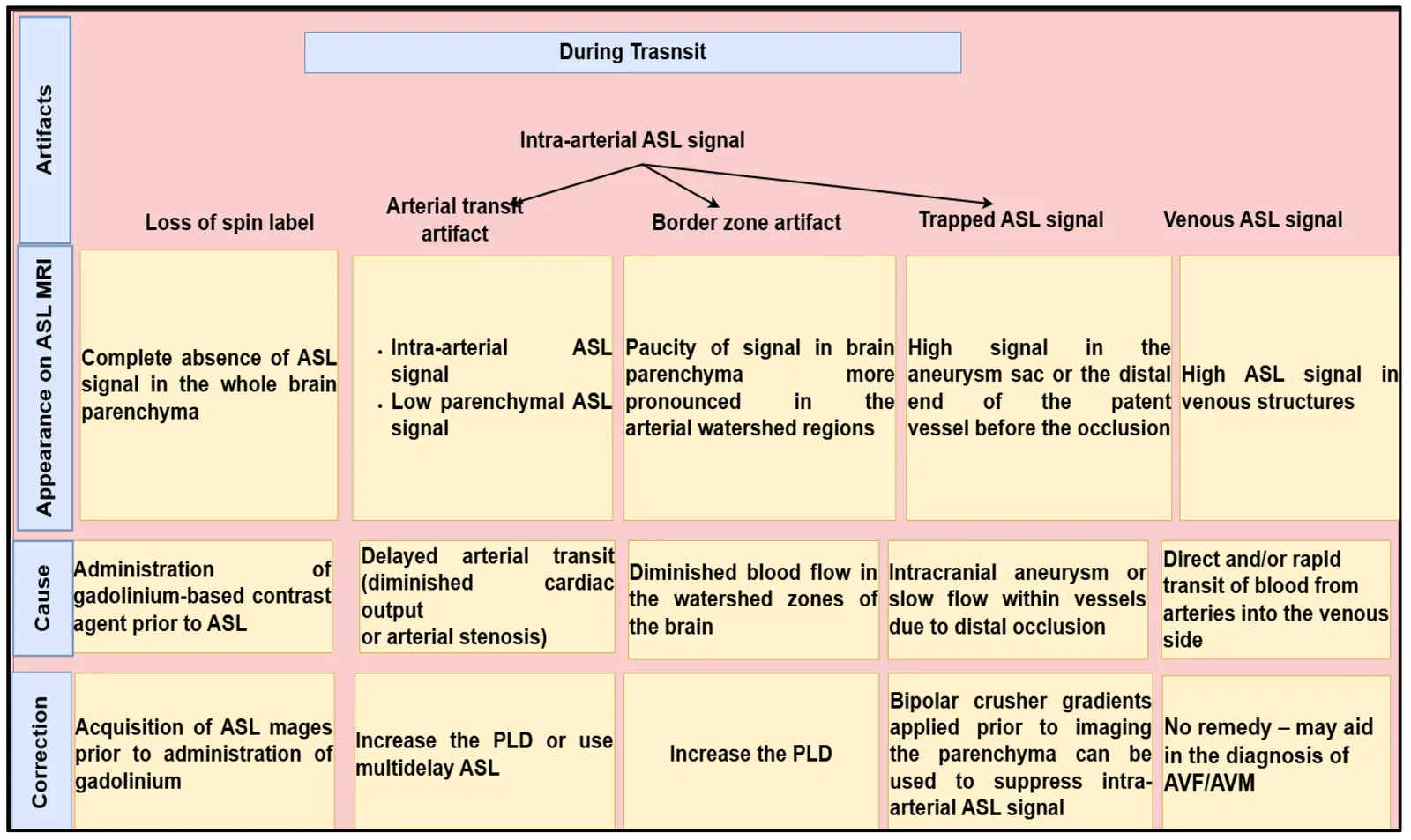
Artifacts occurring during arterial transit of blood in ASL MRI.

**Figure 8:**
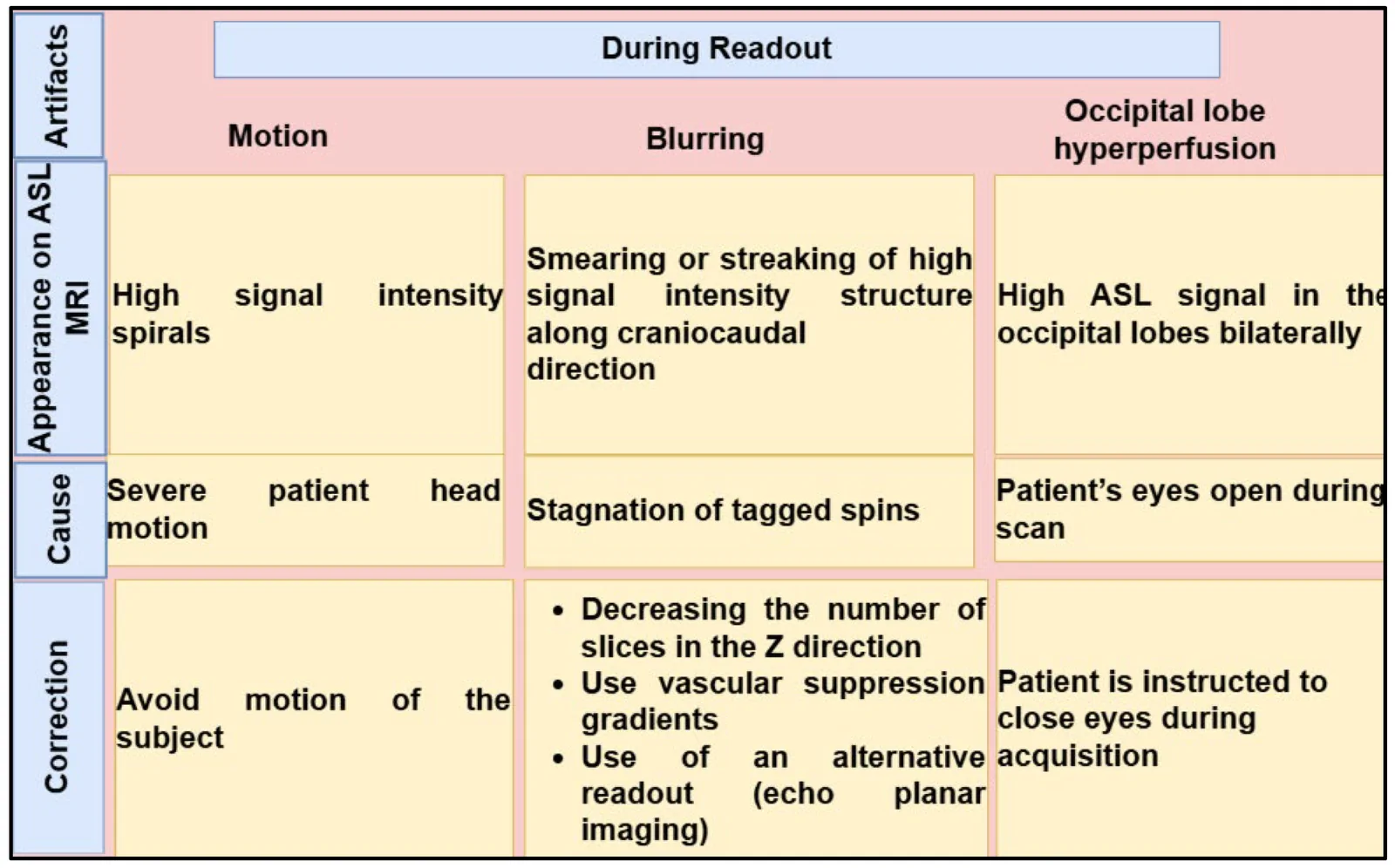
Artifacts occurring during Readout phase in ASL MRI.

Artifacts refer to anomalies or distortions in a signal or data that are not naturally occurring but arise due to limitations or errors in the system. Artifacts in ASL imaging comprise a broader range of undesirable distortions or anomalies that might arise from technical limitations, patient-related factors, or imaging parameters. ASL artifacts can be grouped into three main categories based on when they occur during the imaging process as shown in Figure 5. Artifacts that happen during the labeling of blood, such as ineffective labeling, vessel tortuosity and susceptibility variations near the tagging plane [27,67], are grouped into one category as shown in Figure 6. Artifacts that occur as the labeled blood moves through the brain, including intra-arterial ASL artifact, arterial transit artifact, border zone artifact, trapped ASL signal, and blurring [26], are considered as another category as shown in Figure 7. Artifacts that happen during the actual image capture, such as motion-related artifacts, occipital lobe hyper perfusion, and venous ASL signals, belong to the next category [26,27] as shown in Figure 8. This categorization is crucial for ensuring accurate interpretation of ASL images in clinical studies, as these artifacts can impact the reliability of CBF measurements and diagnostic assessments.

On the other hand, outliers in ASL refer to individual data points that deviate significantly from the expected range, potentially due to measurement errors, physiological variations, or rare pathological conditions [64]. Outliers can be a big issue in ASL MRI due to the relatively low SNR, fluctuations of labeling, the inevitable head motions, and especially the limited number of samples [68]. These outliers can distort the overall perfusion measurements and affect the interpretation of CBF.

The robust fitting method [33] effectively reduces the effect of outliers at the voxel level; however, it overlooks spatial information. Miranda et al. [69] found that motion in unsedated neonates caused artifacts in PWI images and excluded scans with excessive movement to reduce this issue. An M-estimator was created by Maumet et al. [34] to reduce the impact of outliers and increase the robustness of ASL CBF maps. This approach involves clamping the perfusion signal at each voxel before computing the average. Yet, due to its low SNR, the empirical hard threshold may not be accurate in ASL.

Wang et al. [70] developed an outlier detection method that analyzes head motion amplitude and differences using the mean and standard deviation of the brain’s CBF time series. Tan et al. [71] proposed identifying outliers in CBF volumes by excluding those with mean and standard deviation values outside a predefined range, thereby reducing motion and system artifacts. However, these methods rely on reference maps derived from intermediate means, which may already be affected by outliers, and do not account for unaffected voxels within outlier volumes. The Adaptive Outlier Cleaning (AOC) algorithm [35] addresses these issues by independently detecting outlier volumes based on correlation with the mean CBF image. The Structural Correlation-based Outlier Rejection (SCORE) method [36] further enhances AOC by incorporating structural regularization to remove volumes with high spatial correlation to the mean, tackling motion and rotation artifacts. Dolui et al. [36] introduced SCORE+, a similarity-based preprocessing step that is less prone to outliers than mean-difference approaches. The performance of SCORE+ improved even with the elimination of a small number of volumes during the preprocessing step of SCORE+. Yiran Li et al. [37] addressed the issues in the AOC algorithm and introduced a new technique called Priors-guided Slice-wise Adaptive Outlier Cleaning (PAOC) [37], which surpasses existing outlier cleaning algorithms. To better compare the characteristics of the existing artifact and outlier correction methods, Table 4 summarizes their underlying principles, advantages, and limitations.

Although the denoising methods based on DL perform well, the success of this approach heavily relies on the quality of input ASL images. Although deep neural networks are very effective in removing random noise, the existence of strong motion artifacts, labeling issues, and other types of degradation can have different statistics from noise and thereby hinder the process of reconstruction if such degradation is not properly removed during the preprocessing step. Thus, removal of artifacts and outliers remains an important complementary step for enhancing the robustness of AI-driven reconstruction approaches. Moreover, the scarcity of clinical data pairs for training purposes motivated researchers to utilize simulated ASL data for developing algorithms. Such datasets provide a controlled environment for introducing different levels of noise and acquisition-related artifacts, enabling the systematic development and evaluation of robust denoising and reconstruction algorithms.

## 4. ASL Dataset Simulation Procedure

Researchers employed simulated ASL datasets generated from high-resolution structural MRI for experimental purposes, addressing the scarcity of clinical ASL images and the need for a substantial dataset. Various methods have been developed for simulating ASL CBF maps [41,65,66] and L-C pairs [45,49], as described below. Algorithm 1 outlines the fundamental steps for creating high-resolution, noise-free synthetic ASL CBF maps and noisy ASL CBF maps that replicate clinical ASL images. These simulated images serve as reference images and input data, respectively, for denoising algorithms.
**Algorithm 1.** Simulation Procedure for creating synthetic ASL CBF map**Input:** Structural image (T1 MPRAGE) **Output:** ASL CBF map (high resolution and low resolution)i.Segment high-resolution structural MRI (typically from a T1 MPRAGE sequence [72]) using SPM12 [73] software to obtain posteriori probability images or PV estimates of GM $(P_{GM}(x,y,z)),$ WM ($P_{WM}(x,y,z))\;$ and CSF $\left(P_{CSF}(x,y,z)\right)$. Exclude the PV estimation of CSF, as its magnetization is zero.
ii.$\mathrm{Assign}\;a\;\mathrm{CBF}\;\mathrm{value}\;\mathrm{of}\;25\;\mathrm{mL}/100\;g/\min\;\mathrm{to}\;\mathrm{white}\;\mathrm{matter}\;\mathrm{pixels}\;(f_{WM})$, and CBF value of 65 mL/100 g/min to gray matter pixels ($f_{GM})$, based on the reported value for a normal human [41].
iii.Incorporate realistic PV effects [61] into synthetic ASL CBF map using the equation to generate high-resolution noise-free synthetic ASL CBF map f(x,y,z)            $f\left(x,y,z\right)=f_{GM}*P_{GM}\left(x,y,z\right)+f_{WM}*P_{WM}\left(x,y,z\right)\;\;\;\;\;\;\;\;\;\;\;\;\;\;\;\;\;(1)$
iv.Down-sample the high-resolution CBF map, f(x,y,z), to a lower resolution corresponding to a realistic ASL acquisition.
v.Add Gaussian noise at various SNR levels and apply blurring to the synthetic CBF image to simulate clinical conditions [66].

**Figure 9:**
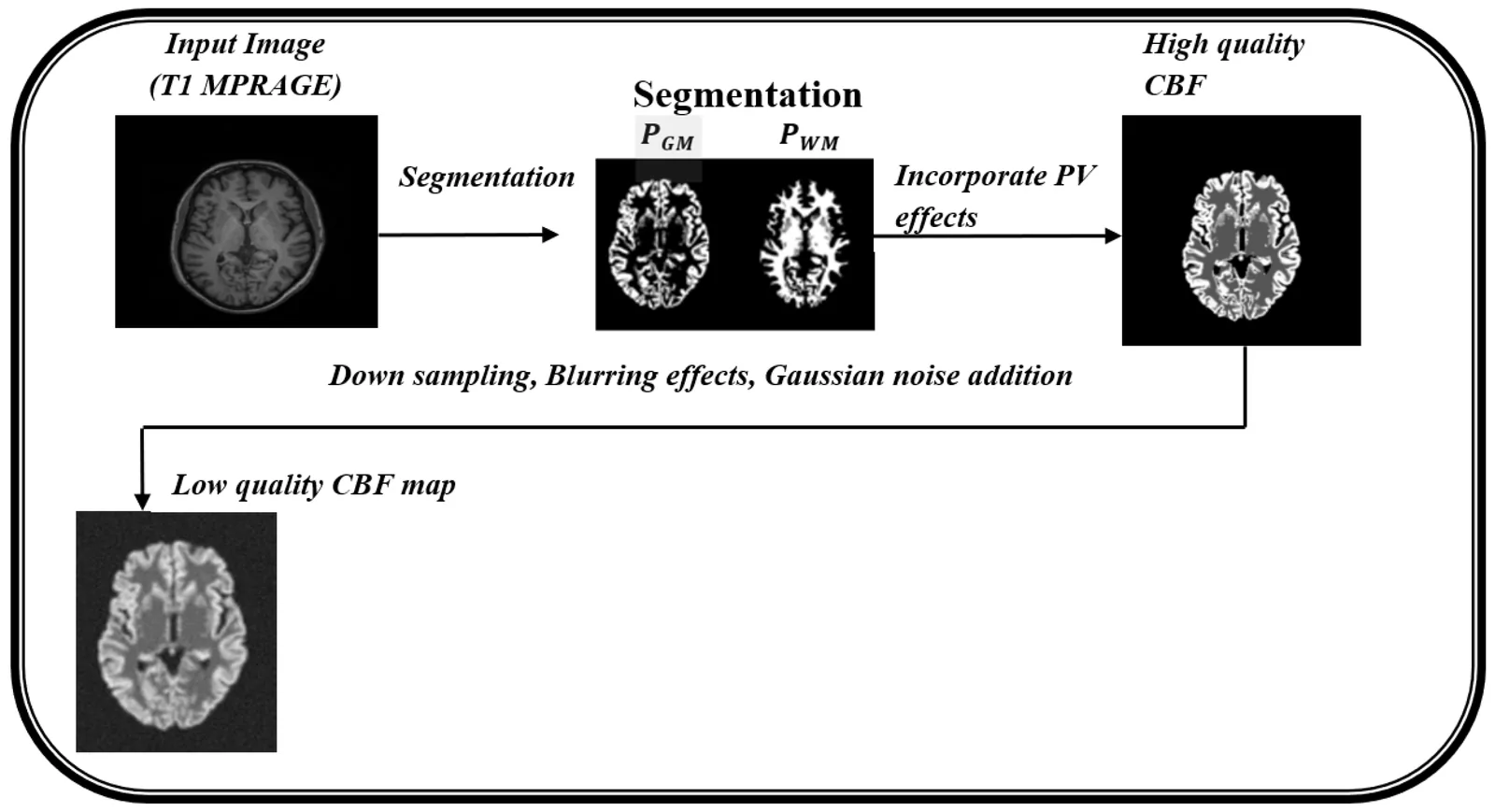
Results of different stages in ASL simulation procedure.

The pictorial results of various steps illustrated in Algorithm 1 are shown in Figure 9.

Step iv in Algorithm 1 can be omitted when PV estimates of GM and WM are directly used in ASL space. These estimates can be obtained by registering the structural images with the control images (or M0 images). Spann et al. [47] employed this method and developed an algorithm to generate ASL L-C pairs from structural and M0 images using a kinetic model. This approach can be applied in situations where researchers aim to reconstruct an ASL CBF map using a minimal number of L-C pairs, especially when real L-C pair images are unavailable. The general procedure for creating the synthetic CBF map, L-C pairs are described in Algorithm 2.
**Algorithm 2.** Simulation Procedure for creating synthetic ASL CBF map and ASL L-C pairs***Input:*** Structural image (T1 MPRAGE), M0 image (PD) ***Output:*** Synthetic ASL CBF map, Label and Control imagesi.$\mathrm{Segment}\;\mathrm{high}-\mathrm{resolution}\;\mathrm{structural}\;\mathrm{MRI}\;(\mathrm{typically}\;T1\;\mathrm{MPRAGE}\;\mathrm{sequence})\;\mathrm{using}\;\mathrm{SPM}12\;\mathrm{software}\;\mathrm{to}\;\mathrm{obtain}\;\mathrm{posteriori}\;\mathrm{probability}\;\mathrm{images}\;\mathrm{or}\;\mathrm{PV}\;\mathrm{estimates}\;\mathrm{of}\;\mathrm{GM}\;(P_{GM}(x,y,z)),\;$ WM ($P_{WM}(x,y,z))$.ii.$\mathrm{Co}-\mathrm{register}\;\mathrm{the}\;\mathrm{tissue}\;\mathrm{PV}-\mathrm{content}\;\mathrm{maps}\;\mathrm{and}\;\mathrm{the}\;T1\;\mathrm{weighted}\;\mathrm{images}\;\mathrm{to}\;\mathrm{the}\;\mathrm{ASL}\;\mathrm{space}\;\mathrm{using}\;M0\;\mathrm{images},\;\mathrm{generating}\;P_{CGM}(x,y,z)),\;$ WM ($P_{CWM}(x,y,z)$.iii.$\mathrm{Assign}\;a\;\mathrm{CBF}\;\mathrm{value}\;\mathrm{of}\;25\;\mathrm{mL}/100\;g/\min\;\mathrm{to}\;\mathrm{white}\;\mathrm{matter}\;\mathrm{pixels}\;(f_{WM})$, and CBF value of 65 mL/100 g/min to gray matter pixels ($f_{GM})$.iv.Incorporate realistic PV effects in synthetic ASL CBF map using the following equation to generate high-resolution noise-free synthetic ASL CBF map f(x,y,z)            $f\left(x,y,z\right)=f_{GM}*P_{CGM}\left(x,y,z\right)+f_{WM}*P_{CWM}\left(x,y,z\right)$Here, $P_{CGM}\left(x,y,z\right)$ and $P_{CWM}\left(x,y,z\right)$ represent the co-registered PV content map of GM and WM respectively.v.Assume Control image as M0 and calculate Label image from Synthetic ASL CBF map f(x,y,z) and Control image using buxton kinectic model [12]            $L\left(x,y,z\right)=c\left(x,y,z\right)-\frac{2\cdot \alpha \cdot M_{0}\left(x,y,z\right)\cdot f\left(x,y,z\right)\cdot {TI}_{1\;\;}\cdot e^{\frac{{T1}_{2}}{{T1}_{b}}}}{\lambda }$vi.Add gaussian noise at different SNR level to each voxel of the synthetic noise free C and L images.vii.Repeat step v *n* times to generate a synthetic data pool containing *n* C/L-pairs.


The simulation procedures described in Algorithms 1 and 2 are primarily intended to generate paired low-quality and high-quality ASL images for image reconstruction tasks, such as denoising and super-resolution, where corresponding clinical image pairs are generally unavailable. The simulation framework follows widely adopted approaches reported in the ASL literature. Specifically, the gray matter and white matter CBF values (65 and 25 mL/100 g/min) are standard physiological reference values for healthy subjects, while the Buxton kinetic model is used as the standard model for ASL signal generation. Similarly, Gaussian noise is introduced because the subtraction of label and control images, each affected by Rician noise, is commonly approximated by a Gaussian distribution, and Gaussian blurring is applied to reproduce the relatively low spatial resolution of ASL images. Although these assumptions provide a controlled and reproducible framework for developing and benchmarking reconstruction algorithms, they do not fully capture clinical variability, including pathology-dependent perfusion changes, ATT heterogeneity, multi-PLD dynamics, and scanner-specific characteristics. Therefore, while simulation-based datasets are valuable for algorithm development, the performance and generalizability of the proposed methods must be validated using independent clinical ASL datasets.

## 5. Challenges in Clinical Translation and Future Research Directions

### 5.1. Challenges in Clinical Translation

Even though significant improvements have been made in the estimation of CBF by using denoising techniques and AI, some limitations remain in the implementation of ASL MRI for clinical purposes. The resolution of these problems is very important to translate the scientific results into clinical practice.

- **Standardization of ASL Protocols:**

Among the critical issues in clinical application of ASL MRI is that there is no standardization of acquisition and processing protocols in different MR scanner vendor models and imaging centers. Differences in magnetic field strength, labeling techniques (PASL, CASL, and PCASL), PLD time, and preprocessing steps lead to differences in CBF quantification, thus hindering reproducibility. Even though the ISMRM Perfusion Study Group released consensus guidelines for ASL acquisition and quantification, they have not yet been implemented uniformly in clinical practice and research studies. Therefore, there is a need for further efforts to achieve acquisition protocol and preprocessing pipeline standardization for better reproducibility and wider clinical adoption.

- **Generalizability of AI Models:**

A number of machine learning and deep learning algorithms have been proposed based on small training and testing datasets sourced from one particular hospital or healthy subjects. Consequently, their performance may differ depending on how they are used for analyzing the images produced by scanners or different imaging modalities. The use of larger and more varied multi-center databases is necessary for training and validating these algorithms in order to increase their clinical usefulness and robustness. Furthermore, deep learning algorithms may generate artifactual or non-physiological patterns of brain tissue perfusion, especially if they have been trained using non-representative or small datasets.

- **Availability of Reliable Reference Data:**

The development of supervised models needs the use of quality CBF maps as references, but the acquisition of such can be challenging since ASL images are naturally noisy and subject to motion and physiological artifacts. Simulated databases, although useful for model training, fail to account for the complexities involved in actual patient data. Hence, more sophisticated simulation systems are needed that would take into account the physiological variation and scanner-specific effects.

- **Clinical Validation**

Most of the AI techniques have shown promising results in improving the quality of images through various quantitative measures such as PSNR and SSIM. However, very limited research has been done on whether these enhancements have any effect on the accuracy of diagnosis or patient treatment or any other clinical significance. Therefore, extensive multi-center clinical trials are needed to verify their clinical importance.

- **Integration into Clinical Workflows:**

Clinical adoption of AI-based ASL techniques hinges on their capacity to deliver consistent and efficient results, seamlessly integrating into the existing clinical imaging workflows, including PACS. In addition, issues related to regulatory approval, data privacy, cybersecurity, and quality assurance must be addressed to ensure safe and practical deployment in healthcare environments.

- **Interpretability of AI Models:**

Although numerous deep learning models have proved to be very promising in terms of their performance capabilities, most of them have been labeled as “black box,” making it difficult for physicians to understand the reasoning behind the output obtained from the model. Incorporation of Explainable Artificial Intelligence (XAI)-based approach within deep learning models can help in achieving better transparency through interpretation.

- **Integrated Artifact and Outlier Correction:**

Even though AI algorithms have demonstrated great efficiency in denoising images in ASL, artifact detection and correction along with the outliers’ removal continue to rely mostly on traditional image-processing techniques. Future research is needed to create AI solutions that would be capable of detecting artifacts and outliers, denoising images, and estimating the value of CBF simultaneously.

### 5.2. Future Research Directions

Future research in ASL imaging is expected to focus on the development of advanced preprocessing methods and AI-driven techniques to achieve more accurate CBF quantification, enhanced image quality, and improved clinical applicability.

- **Enhancing Preprocessing for CBF Quantification**

Future work should focus on improving the ASL image preprocessing pipeline [68] and evaluating its impact on the generation of CBF maps for training deep learning models. In addition, evaluating recently developed outlier detection methods as alternatives to SCORE+ [36] and exploring multivariate machine learning approaches for CBF quantification may contribute to improved accuracy and robustness.

- **Improving Deep Learning Architectures**
  ➢ Existing models, such as the Dual Independent Pathway–Densely Connected Residual Network with Dilated Convolution (DIP-DRDC), can be further enhanced through improvements in network architecture, training strategies, and validation methodologies. Training these models using better datasets tailored towards ASL imaging can produce favorable results.
  ➢ Generative models, particularly Generative Adversarial Networks (GANs) [74,75], offer a promising approach for generating synthetic L–C pairs, helping to address the limited availability of training datasets while also reducing acquisition time. However, despite these advantages, it is essential to verify the accuracy of the generated images. If the training dataset is not sufficiently representative, the resulting synthetic images may contain unrealistic perfusion characteristics, which could introduce bias into CBF measurements.
  ➢ The use of attention mechanisms [76] in models such as DIP-DRDC may allow the network to assign greater importance to relevant features while reducing the influence of irrelevant or noisy features. Moreover, incorporating TV regularization into the loss function may help ensure an appropriate balance between data fidelity and regularization in the generated images.
- **Fine-Tuning and Longitudinal Studies**

Training models on diverse datasets that include data from different patient populations and imaging protocols may improve their clinical applicability and generalizability. Furthermore, longitudinal studies may help evaluate the ability of the model to detect changes in CBF over time and monitor disease progression as well as treatment response.

- **Functional Applications and ASL fMRI Integration**

The extension of the DIP-DRDC framework to ASL-based fMRI may facilitate the study of brain functional connectivity. Furthermore, incorporating denoising techniques into the model could improve the accuracy of functional connectivity assessment.

- **Optimization Techniques for Multi-PLD Approaches**

Future research could focus on identifying the optimal set of PLDs for multi-PLD ASL. Reducing the number of PLDs required to generate accurate CBF and Arterial Transit Time (ATT) maps has the potential to decrease scan time without compromising image quality. This would improve the feasibility of ASL MRI in clinical settings, where shorter acquisition times are particularly beneficial.

Designing genetic algorithms for the development of optimization techniques to find the optimum number of PLDs is an important area of research. This technique can help in finding the best possible configuration of PLDs that guarantees proper calculation of CBF and ATT. In addition, advanced encoding strategies such as Hadamard-encoded ASL, time-encoded ASL, and other optimized labeling schemes [77] offer a promising direction for accelerating multi-PLD acquisitions by improving SNR efficiency and enabling the acquisition of multiple effective PLDs within a single scan. Combining these encoding strategies with deep learning-based reconstruction and optimization techniques may further enhance image quality while reducing overall scan time.

- **Domain Adaptation and Multi-center Generalization**

Currently, most ASL image enhancement models are trained and validated using data from a single institution or only a few MRI scanners. This limited diversity can reduce their performance when they are applied to images acquired using different scanners or imaging protocols. Developing robust domain adaptation methods is therefore an important research direction, as these approaches can improve the ability of models to perform consistently across a wide range of clinical settings.

- **AI-based techniques for artifacts and Outlier detection and removal**

Although AI-based denoising methods have demonstrated encouraging results for ASL image enhancement, the application of ML and DL to artifact and outlier detection remains relatively limited. One of the major challenges is the large variability in artifact patterns and outlier characteristics across different ASL datasets, making it difficult to develop models that generalize well to diverse clinical data. As a result, conventional correction methods are still widely used in practice, and the images generated using these techniques are often employed as reference data for training deep learning models.

Future research could focus on developing AI-based methods capable of automatically identifying and correcting artifacts and outliers in ASL images. Because well-annotated clinical datasets are scarce, the use of synthetic or augmented data may provide an effective way to support model development and validation.

Advances in these areas are expected to improve the robustness of ASL image analysis and promote the wider clinical adoption of ASL imaging.

## 6. Conclusions

ASL MRI holds immense potential as a noninvasive and patient friendly modality for CBF quantification, offering significant benefits over traditional imaging techniques by eliminating the need for external contrast agents. This review examined the recent progress in ASL MRI by addressing four research questions (RQ1–RQ4). The evidence reviewed for RQ1 shows that image quality and CBF estimation have improved considerably with the evolution of traditional image processing methods, ML, and DL techniques. Among these, DL has shown particular promise in producing high-quality CBF maps while reducing the number of L–C pairs required for reconstruction, thereby contributing to shorter acquisition times. RQ2 highlighted that effective artifact and outlier correction remains essential for obtaining reliable CBF measurements, although further development of AI-based approaches is needed. RQ3 demonstrated the importance of simulation frameworks in overcoming the limited availability of annotated ASL datasets for training and validating DL models. Finally, RQ4 showed that advancements in imaging techniques, preprocessing, and computational methods are essential to improving image quality, minimizing scan duration, increasing reproducibility, and expanding clinical use of ASL MRI. The findings and future directions discussed in this review are intended to support ongoing research aimed at improving the accuracy, efficiency, and clinical applicability of ASL MRI for brain perfusion imaging.

## Figures and Tables

**Table 1:** Comparison of existing ASL review articles.

Reference	Primary Focus	AI-Based Denoising	Artifact & Outlier Correction	Simulation Frameworks	Clinical Translation	Unique Contribution
Petcharunpaisan et al. [14]	Fundamentals of ASL and neuroimaging applications	✗	✗	✗	Partial	Introduced ASL principles and neuroimaging applications.
Clement et al. [17]	ASL image processing pipeline for beginners	✗	Partial	✗	✗	Focused on preprocessing and image processing workflow.
Lindner et al. [18]	ISMRM clinical guidance for ASL MRI	✗	✗	✗	✓	Standardization, acquisition recommendations, and clinical implementation.
Woods et al. [20]	Multi-timepoint ASL acquisition and quantitative perfusion	✗	✗	✗	✓	Recommendations for multi-PLD ASL acquisition and quantification.
Sollmann et al. [15]	Methodological overview and clinical applications	✗	✗	✗	✓	Overview of ASL methodology and clinical use cases.
Chappell et al. [16]	Partial volume correction	✗	✗	✗	Partial	Comprehensive review of PVE correction techniques.
Tanaka et al.[10], Telischak et al. [21], Iutaka et al. [22], Jaafar et al. [23], Togao et al. [24]	Clinical applications of ASL MRI	✗	✗	✗	✓	Disease-specific and clinical applications.
Jaganmohan et al. [26]	ASL artifacts and remedies	✗	✓	✗	Partial	Comprehensive review of ASL artifacts and their correction.
Amukotuwa et al. [27]	Clinically significant ASL artifacts	✗	✓	✗	Partial	Review of clinically significant ASL artifacts.
Alsaedi et al. [25]	General overview and critical appraisal of ASL	✗	Partial	✗	Partial	General overview of ASL techniques and challenges.
Proposed Review	AI-enhanced ASL image reconstruction for CBF quantification	✗	✓	✓	✓	Comprehensive review of denoising, artifact/outlier correction, simulation frameworks, and clinical translation organized around RQ1–RQ4.

Note: ✓ indicates that the corresponding feature is supported or present; ✗ indicates that the corresponding feature is not supported or absent.

**Table 2:** Conceptual summary of noise reduction strategies in ASL MRI.

Category	Method/Principle	Advantages	Limitations
Physiological noise correction	GLM [38], CompCor [39], RETROICOR [40]	Model or regress physiological fluctuations, improve perfusion reliability	Assumptions may not hold; residual noise may remain
Spatial filtering	Gaussian [41], Wavelet [42], ICA [42], Anisotropic diffusion [42]	Improve SNR, reduce random/systematic noise	May smooth edges, alter quantitative CBF
Temporal filtering	Adaptive NLM [43], DT-CWT [44]	Utilize temporal redundancy, preserve connectivity in ASL-fMRI	Can introduce temporal blurring, loss of high-frequency info
Spatio-temporal methods	TV regularization [45], STLRTV [46], TGV [47], PCA [48], NESMA [49]	Balance denoising with edge preservation, robust to artifacts	Require larger datasets, computationally intensive
Machine Learning	SVMASLQ [50], Patch-wise SVM [51]	Exploit spatial correlations, improve local detail	Computationally expensive, sensitive to parameter choice
Deep Learning	Dilated Conv [52], Residual CNN [53], ASLDLD [54], Deep convolutional joint filter [55], Denoising Autoencoder [56], Unsupervised network [57], DWAN [58], DIP-DRDC ASL [59], HUST [60]	Learn complex spatio-temporal patterns, strong denoising with fewer L–C pairs, superior image quality	Data-hungry, needs reference images or augmentation, generalizability remains a challenge

**Table 3:** Studies conducted in ASL MRI on enhancing the CBF quantification.

Category	Technique Used	Labeling Technique	Single/Multiple (PLD/TI)	Number of L–C Pairs	Dataset	Performance Evaluation Metric	Key Observations/Limitations
Physiological noise reduction	General linear model (GLM) [38]	PICORE-QUIPSS II	Single TI	130	In vivo, 7 subjects (Visual cortex), 7 subjects (Hippocampus)	*p* values, F-statistics, multiple correlation coefficients	▪ Reduced physiological fluctuations but residual noise remained. ▪ Depends on assumptions about the nature and contribution of physiological noise.
	CompCor using PCA [39]	PICORE-QUIPPS II	Single TI	NA	In vivo, 10 subjects	Paired t-tests, Spectral analysis, Band-averaged coherence, Cohxy (fband), ROC curve, tSTD	▪ No need for external physiological monitoring (cardiac/respiratory data not required). ▪ Limited effectiveness when noise is highly correlated with the signal of interest.
	RETROICOR [40]	PASL FAIR and CASL	Single TI	60	In vivo, 10 subjects	Two-tailed paired Student’s *t*-test, Temporal SNR	▪ Corrects both global and localized physiological noise. ▪ Combination with linear deconvolution addresses both synchronized and non-synchronized physiological noise. ▪ Effectiveness depends on signal periodicity; irregular fluctuations may not be well corrected.
Spatial noise reduction	Wavelet-based filtering [41]	PICORE Q2TIPS	Single TI	300, 40	Simulated, CBF from structural images (GM = 65, WM = 20 mL/100 g/min), 3 subjects	Std deviation, Coefficient of variation, SNR	▪ Wavelet better than Gaussian. ▪ Affects absolute CBF values near tissue borders and edges, leading to potential inaccuracies in those regions. ▪ Not uniformly reliable across all regions, limiting precision in specific image analyses.
	Wiener, Anisotropic diffusion, Gaussian, Wavelet decomposition, ICA [42]	CASL (Animal), PCASL GRASE (Human)	Multiple PLD, Single PLD	5, 64	Simulated, CBF = 250 mL/min/100 g for 12 PLDs; in vivo 3 male rats, In vivo, single subject	CNR, absolute difference from gold standard	▪ Showed effectiveness in reducing both random and systematic errors. ▪ ICA minimized bias while preserving image contrast. ▪ Customization needed on a subject-specific basis, limiting general applicability.
Temporal noise reduction	Non-Local Means (NLM) [43]	PASL EPISTAR with Look-Locker	Multi-TI	150	Simulated (fitted from healthy volunteer)	SNR	▪ Clear image quality improvements compared to Gaussian smoothing and standard spatial NLM filtering. ▪ Valuation used only SNR as a metric, lacking comprehensive validation.
	Dual-tree complex wavelet transform (DT-CWT) [44]	3D-GRASE pCASL	Single PLD	59	Simulated/in vivo, 10 subjects	SNR, Sensitivity, Accuracy	▪ Reduces noise while preserving spatial features important for functional connectivity analysis. ▪ Effective in low-SNR conditions, allowing useful results even with limited time points. ▪ Introduces significant temporal smoothing, which can suppress high-frequency image features.
Spatio-temporal noise reduction	Spatio-Temporal Low Rank Total Variation [45]	PCASL	Single PLD	128	In vivo, 2 subjects	PSNR	▪ Superior noise reduction compared to existing post-processing methods. ▪ Recovers ASL sequences even from heavily corrupted data. ▪ Balances denoising with edge preservation. ▪ No direct comparison with other advanced spatial or temporal filtering approaches. ▪ Effectiveness not benchmarked across a wide range of competing methods.
	Two-step 3D Total Variation regularization [46]	PCASL	Single PLD	NA	In vivo, 5 subjects	CNR	▪ Reduces noise effectively in both temporal and spatial domains. ▪ Preserves important image features (edges, structures). ▪ Dual-step approach increases robustness compared to single-domain filtering. ▪ Requires many L–C pairs, which increases acquisition demands. ▪ Less practical in resource-constrained settings or when fewer L–C pairs are available.
	Total Generalized Variation (TGV) [47]	PICORE-Q2TIPS	Single TI	500, 100	In vivo, 10 subjects	PSNR, SSIM	▪ Outperformed other methods in edge preservation. ▪ Preserves structural details in complex regions.
	Robust PCA [48]	PCASL, 2D EPI	Single PLD	40	In vivo, 43 subjects	TSNR	▪ Enhanced TSNR. ▪ Computationally intensive, since RPCA decomposition is costly for large ASL datasets. ▪ Performance may be sensitive to the choice of decomposition thresholds.
	NESMA [49]	PCASL	Single PLD	3, 5, 10, 20, 30	Simulated (noise-free control, labeled, PD images) + in vivo, 10 subjects	Bias, Dispersion	▪ Preserved fine structures, reduced bias and dispersion. ▪ Performance may depend on quality and consistency across modalities. ▪ Potential sensitivity to registration errors between different acquisition types.
Machine Learning	SVMASLQ [50]	CASL	Single PLD	50	Simulated, synthetic baseline; In vivo, 13 healthy, 2 patients (moyamoya & Alzheimer’s)	SNR	▪ Improved spatial SNR, limited local adaptation. ▪ Computationally expensive.
	Patch-wise SVM [51]	PCASL	Single PLD	40	In vivo, 30 subjects	SNR	▪ Better local suppression; computationally expensive. ▪ Sensitive to patch size selection, which affects performance.
Deep Learning	Dilated Conv [52]	PCASL (Conventional & Hadamard)	Single PLD	6	In vivo, 7 healthy, 114 patients (tumor, infarction, Moyamoya)	MSE, Bland–Altman, Wilcoxon test, Radiologist eval	▪ Outperforms averaging. ▪ Extended training time. ▪ Subjective image quality.
	CNN residual learning [53]	PCASL	Single PLD	30	Simulated, 5 subjects	PSNR, RMSE, Lin’s CCC, Bland–Altman	▪ Superior CBF estimation. ▪ Relies on clean reference images.
	ASLDLD [54]	PCASL	Single PLD	40	In vivo, 240 subjects	SNR	▪ Outperforms non-deep learning methods. ▪ No direct comparison with other DL methods.
	Deep convolutional joint filter [55]	PCASL	Single PLD	30	In vivo, 35 subjects	PSNR	▪ Effective denoising & artifact correction. ▪ Uses filtered reference images.
	Unsupervised learning framework [56]	PCASL	Single PLD	NA	In vivo, 3 subjects	PSNR, SSIM, Wilcoxon test	▪ Promising results for limited dataset. ▪ Potential improvement in network design needed.
	Denoising autoencoder [57]	PCASL	Single & Multi-PLD	10	In vivo, 135 patients	PSNR, SSIM	▪ Improved image clarity. ▪ Requires broader validation and comparison with DL models.
	DWAN [58]	PCASL	Single PLD	40	In vivo, 280 subjects	PSNR, SSIM, Lin’s CCC, Bland–Altman	▪ High denoising performance across noise levels. ▪ Limited generalizability to pathological conditions.
	DIP-DRDC ASL [59]	PASL	Single PLD	52	In vivo, 72 healthy, 30 patients (AD)	PSNR, SSIM, Lin’s CCC, Bland–Altman	▪ Outperforms previous methods. ▪ Subjective evaluation across various clinical scenarios needed.
	HUST [60]	PCASL	Single PLD	NA	2D, Healthy, 277 3D, 110	PSNR, SSIM	▪ Outperforms existing denoising methods by preserving image texture. ▪ Requires validation across various clinical scenarios and different MRI acquisition protocols.

**Table 4:** Overview of studies conducted in ASL MRI to improve artifacts and outlier correction.

Method	Working Principle	Advantages	Limitations
Robust Fitting [33]	Reduces the influence of voxel-wise outliers during CBF estimation.	Simple implementation and effective in suppressing isolated outliers.	Does not consider spatial relationships between neighboring voxels, limiting performance in low-SNR images.
Motion Exclusion [69]	Discards scans affected by excessive head motion.	Effectively removes severe motion-corrupted images.	Reduces the number of available L–C pairs, which may decrease SNR.
M-estimator [34]	Reduces the influence of extreme voxel values before averaging.	Improves robustness of CBF estimation in the presence of outliers.	Performance depends on empirically selected thresholds and may be less reliable for very noisy ASL data.
Mean/Standard Deviation-based Detection [70,71]	Detects abnormal CBF volumes using statistical thresholds.	Fast and computationally efficient.	Reference statistics may themselves be affected by outliers, leading to misclassification.
AOC [35]	Identifies outlier volumes using correlation with the mean CBF image.	More adaptive than simple statistical thresholding methods.	Performance depends on the quality of the reference image and selected correlation thresholds.
SCORE [36]	Combines outlier detection with structural MRI information.	Improves removal of motion-related artifacts while preserving anatomical information.	Requires accurate image registration and reliable structural segmentation.
SCORE+ [36]	Similarity-based enhancement of SCORE.	More robust against outliers and improves CBF quality with minimal data loss.	Validation across different scanners and disease populations remains limited.
PAOC [37]	Improved adaptive outlier cleaning approach.	Better preserves useful perfusion information while removing corrupted volumes.	Computationally more demanding and requires broader clinical validation.

## Data Availability

No new data were created or analyzed in this study. Data sharing is not applicable to this article. All information and data discussed in this review are contained within the article and are based on previously published and appropriately cited literature.

## Abbreviations

The following abbreviations are used in this manuscript.

AD	Alzheimer’s Disease
ADNI	Alzheimer’s Disease Neuroimaging Initiative
AI	Artificial Intelligence
AOC	Adaptive Outlier Cleaning
ANTs	Advanced Normalization Tools
ASL	Arterial Spin labeling
ASLDLD	Arterial Spin Labeling Deep Learning Denoising
ATT	Arterial Transit Time
CCC	Concordance Correlation Coefficient
CASL	Continuous ASL
CBF	Cerebral Blood Flow
CNN	Convolutional Neural Networks
CNR	Contrast to Noise Ratio
CompCor	Component-Based Noise Correction Method
DIP-DRDC	Dual Independent Pathway–Densely Connected Residual Network with Dilated Convolution
DL	Deep Learning
DT-CWT	Dual-tree complex wavelet transform
DWAN	Deep Weighted Averaging Network
EPISTAR	Echo Planar Imaging and Signal Targeting with Alternating Radio Frequency
FAIR	Flow-sensitive Alternating Inversion Recovery
fMRI	Functional MRI
FSL	Functional Magnetic Resonance Imaging of the Brain Software Library
GAN	Generative Adversarial Networks
GLM	General Linear Model
GM	Gray Matter
GRASE	Gradient and Spin Echo
ICA	Independent Component Analysis
ISMRM	International Society for Magnetic Resonance in Medicine
L-C	Label–Control
ML	Machine Learning
MPRAGE	Magnetization-Prepared Rapid Gradient Echo
MRI	Magnetic Resonance Imaging
MSE	Mean Squared Error
NESMA	Non-local Estimation of Multispectral Magnitudes
NLM	Non-Local Means
PAOC	Priors-guided Slice-wise Adaptive Outlier Cleaning
PASL	Pulsed Arterial Spin Labeling
PCA	Principal Component Analysis
PCASL	Pseudo-Continuous Arterial Spin Labeling
PD	Proton Density
PICORE	Proximal Inversion with a Control for Off-Resonance Effects
PLD	Post-Labeling Delay
PV	Partial Volume
PVE	Partial Volume Effect
PSNR	Peak SNR
PWI	Perfusion-Weighted Image
Q2TIPS	QUIPSS II with Thin-slice TI_1_ Periodic Saturation
QUIPSS	Quantitative Imaging of Perfusion using a Single Subtraction, Second Version
RF	Radio Frequency
RETROICOR	RETROspective Image CORrection
RMSE	Root Mean Square Error
ROC	Receiver Operating Characteristic
RPCA	Robust Principal Component Analysis
SAR	Specific Absorption Rate
SCORE	Structural Correlation-based Outlier Rejection
SNR	Signal to Noise Ratio
SPM	Statistical Parametric Mapping
STLRTV	Spatio-Temporal Low-Rank Total Variation
SVM	Support Vector Machine
SVMASLQ	Support Vector Machine-based Arterial Spin Labeling Quantification
TGV	Total Generalized Variation
TI	Inversion Time
TV	Total Variation
tSNR (TSNR)	Temporal Signal-to-Noise Ratio
WM	White Matter
XAI	Explainable Artificial Intelligence
